# Impact of Acetazolamide and CPAP on Cortical Activity in Obstructive Sleep Apnea Patients

**DOI:** 10.1371/journal.pone.0093931

**Published:** 2014-04-07

**Authors:** Katrin Stadelmann, Tsogyal D. Latshang, Yvonne Nussbaumer-Ochsner, Leila Tarokh, Silvia Ulrich, Malcolm Kohler, Konrad E. Bloch, Peter Achermann

**Affiliations:** 1 Institute of Pharmacology and Toxicology, University of Zurich, Zurich, Switzerland; 2 Zurich Center for Integrative Human Physiology, University of Zurich, Zurich, Switzerland; 3 Pulmonary Division, University Hospital Zurich, Zurich, Switzerland; 4 University Hospital of Child and Adolescent Psychiatry and Psychotherapy, University of Bern, Bern, Switzerland; University of Oxford, United Kingdom

## Abstract

**Study Objectives:**

1) To investigate the impact of acetazolamide, a drug commonly prescribed for altitude sickness, on cortical oscillations in patients with obstructive sleep apnea syndrome (OSAS). 2) To examine alterations in the sleep EEG after short-term discontinuation of continuous positive airway pressure (CPAP) therapy.

**Design:**

Data from two double-blind, placebo-controlled randomized cross-over design studies were analyzed.

**Setting:**

Polysomnographic recordings in sleep laboratory at 490 m and at moderate altitudes in the Swiss Alps: 1630 or 1860 m and 2590 m.

**Patients:**

Study 1: 39 OSAS patients. Study 2: 41 OSAS patients.

**Interventions:**

Study 1: OSAS patients withdrawn from treatment with CPAP. Study 2: OSAS patients treated with autoCPAP. Treatment with acetazolamide (500–750 mg) or placebo at moderate altitudes.

**Measurements and Results:**

An evening dose of 500 mg acetazolamide reduced slow-wave activity (SWA; approximately 10%) and increased spindle activity (approximately 10%) during non-REM sleep. In addition, alpha activity during wake after lights out was increased. An evening dose of 250 mg did not affect these cortical oscillations. Discontinuation of CPAP therapy revealed a reduction in SWA (5–10%) and increase in beta activity (approximately 25%).

**Conclusions:**

The higher evening dose of 500 mg acetazolamide showed the “spectral fingerprint” of Benzodiazepines, while 250 mg acetazolamide had no impact on cortical oscillations. However, both doses had beneficial effects on oxygen saturation and sleep quality.

## Introduction

Obstructive sleep apnea syndrome (OSAS) is a highly prevalent respiratory disorder characterized by recurrent episodes of partial or complete collapse of the upper airways during sleep [1], affecting 3–7% of the population [2]. An established and effective therapy for OSAS is treatment with continuous positive airway pressure (CPAP), which prevents obstructive apneas, stabilizes sleep [3], [4] and reduces daytime symptoms [5]. It is known that OSAS patients show alterations in brain activity, for example a slowing of the EEG during waking compared to healthy controls [6], [7], as well as reduced slow-wave activity (SWA), theta and sigma activity and slowing of sleep spindles during sleep [8]–[11]. The efficacy of CPAP treatment is reflected in the normalization of cortical activity after several months, as indicated by increased SWA during non-REM sleep and absent EEG slowing during wakefulness [6], [12].

Since mountain tourism has increased during the last decades, it is nowadays popular to spend weekends and holidays at mountain resorts or lodges located at moderate altitudes between 1500 and 3000 m. Sleep at altitude is altered in healthy individuals. A shift towards lighter sleep together with an increase in central apneas has been observed at altitude [13]–[16]. Compared to the obstructive apneas observed at baseline in OSAS patients, central apneas induced by an ascent to altitude are characterized by the intermittent absence of the drive to breathe [17] and are generated by the brainstem respiratory center as a response to changes in blood gas concentrations [18]. One treatment for central apneas at altitude is acetazolamide, a carbonic anhydrase inhibitor frequently used in the treatment of acute mountain sickness [19], [20]. Acetazolamide prevents central apneas at altitude through metabolic acidosis by its diuretic effects [21]. Sleep of OSAS patients at 490 m and at 1630 to 2590 m with and without CPAP treatment was investigated in three previous studies [3], [22], [23], in which acetazolamide was shown to reduce central apneas compared to placebo during sojourns to moderate altitude [3], [22]. In addition, as observed in healthy subjects [24], acetazolamide in OSAS patients reduced the apnea/hypopnea index compared to placebo and increased oxygen saturation and improved sleep quality (sleep efficiency, arousals, slow-wave sleep) [3], [22].

While the positive effect of acetazolamide on breathing and sleep architecture at altitude has been investigated [19], its effect on cortical oscillations remains unknown. Indeed, acetazolamide is known to reduced cortical excitability and for this reason has been used in the treatment of epilepsy [25].

A primary aim of the present study was to examine the effect of acetazolamide on cortical activity as measured by the sleep and wake EEG at moderate altitude in OSAS patients. We used quantitative analysis of the sleep and wake EEG, namely spectral analysis, to quantify the changes in cortical activity. Due to its frequent use and beneficial effect on sleep quality, the impact of acetazolamide on brain function is of great interest. As some patients discontinue their CPAP therapy for short time periods for various reasons a secondary aim of this study was to investigate changes in cortical oscillations during non-REM sleep resulting from short-term discontinuation of CPAP therapy in previously CPAP-treated OSAS patients.

## Materials and Methods

**Figure 1:**
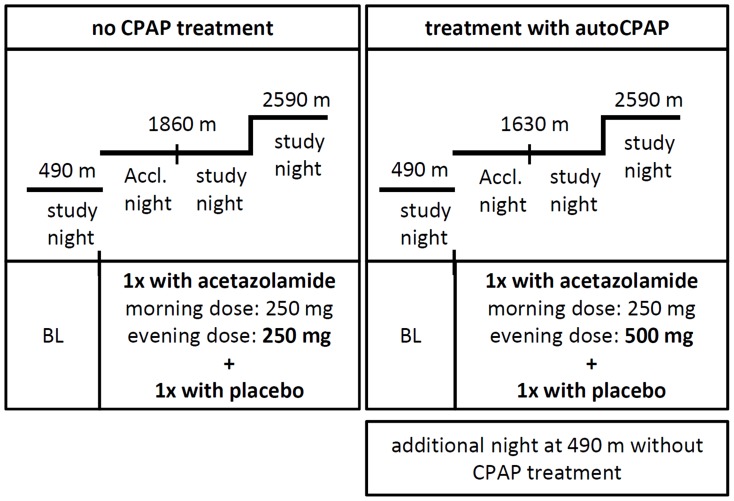
Design of the two studies (Study 1 [16]; Study 2 [3]). The n indicates the number of participants included in the present analyses. In both studies, patients underwent two 3-day sojourns at two moderate altitudes, once receiving acetazolamide and once receiving placebo with a 2 week washout period at baseline level (BL, 490 m). The first night at altitude (1860 m in study 1 and 1630 m in study 2) always served for acclimatization. The order of altitude exposure of the two study nights at lower (1860 m and 1630 m) and higher altitude (2590 m) and at baseline (BL, 490 m) were performed in a randomized cross-over design with regard to the order of altitude exposure. Patients in study 1 stopped CPAP treatment starting 3 nights before study periods at altitude and baseline. In total 5 study nights were analyzed in study 1 and 6 in study 2.

We analyzed data from two randomized, placebo-controlled, double-blind crossover trials [3], [22] to evaluate the effect of two doses of acetazolamide on the non-REM sleep and wake EEG spectra in OSAS patients with and without CPAP treatment. In both studies patients underwent baseline recordings at 490 m and two study nights at moderate altitudes, during which they received acetazolamide and placebo (see Figure 1; for study protocols see Latshang et al. [3] and Nussbaumer-Ochsner et al. [22]). The two studies were conducted in Zurich, Switzerland (baseline) and Davos, Switzerland (moderate altitude) according to a similar study protocol [3], [22]. Patients were studied during 2 sojourns of 3 days at moderate altitude, 2 days (one acclimatization day) at 1860 m (study 1) or 1630 m (study 2), 1 day at 2590 m (both studies), separated by a 2-week washout period at low altitude (<800 m). The studies are characterized by two primary methodological differences (see Figure 1): 1) Patients in study 1 [22] stopped CPAP treatment starting 3 nights before study periods at altitude and baseline and received no CPAP during the study (we refer to this condition as ‘no CPAP’). On the other hand, study 2 participants were treated with autoCPAP (mask pressure 5–15 cm H_2_O [3]; referred to as ‘with CPAP’). 2) The dose of acetazolamide administered in the evening was twice as high in study 2 [3] compared to study 1 (Nussbaumer-Ochsner et al. [22]; 500 mg compared to 250 mg). In both studies, 250 mg of acetazolamide was administered in the morning. In addition, patients in study 2 performed an additional recording at 490 m on the last of 4 nights of CPAP withdrawal to assess the effect of short-term CPAP withdrawal [3].

### Participants

Male and female patients with OSAS receiving long-term CPAP treatment, living at low altitude (<800 m) participated in the study. All participants had a prior diagnosis of OSAS based on excessive sleepiness and an elevated obstructive apnea/hypopnea index (AHI; >10/h) with predominant obstructive events prior to initiation of CPAP treatment. Thirty-nine of 49 subjects in study 1 (mean age (SD): 62 (7.6) years; obstructive AHI in the range of 16/h to 90/h at 490 m, Table S1 in File S1) and 41 of 51 subjects in study 2 (60 (8.5) years; obstructive AHI 18/h to 94/h at 490 m, Table S2 in File S1) were included in the present analysis. The exclusion criteria for EEG analyses were insufficient quality of the non-REM sleep EEG spectra due to artifacts. Details on demographics, study designs and inclusion/exclusion criteria have been reported previously [3], [22].

### Ethics Statement

Both studies were approved by the ethics committee of the Canton of Zurich (Switzerland) and patients gave their written informed consent. The studies were registered (clinicaltrials.gov; ID#NCT00714740 and ID#NCT00928655).

### Polysomnographic recordings

At each session, nighttime sleep was polysomnographically recorded with Alice5 (Philips Respironics AG, Zofingen, Switzerland). The EEG (derivation C3A2), submental EMG, EOG and respiratory signals consisting of calibrated inductance plethysmography, nasal pressure swings and pulse oximetry were measured [3], [22]. The EEG was sampled at 200 Hz (high-pass filter at 0.32 Hz; low-pass filter at 100 Hz; notch filter at 50 Hz). Sleep stages (30-s epochs) and arousals were visually scored according to standardized criteria [17], [26].

### Quantitative EEG analysis

Spectral analysis was performed on consecutive 30-s epochs (FFTW approach, Hanning window, averages of six 5-s epochs; frequency resolution 0.2 Hz). The three lowest frequency bins (0.2–0.6 Hz) were excluded from analysis because of their sensitivity to low frequency artifacts. Spectral data were analyzed up to 20 Hz.

Non-REM sleep EEG power density spectra were calculated over the minimal common length of non-REM sleep within individuals. REM sleep EEG spectra could not be analyzed as subjects in study 1 (no CPAP) had insufficient amounts of REM sleep due to frequent apneas. Average wake EEG power density spectra were determined over all available epochs per night (i.e. after lights out), if all five study nights included at least 20 artifact-free 30-s epochs of waking. A wake EEG analysis was performed for 30 (of 39 in study 1) and 33 subjects (of 41 in study 2). Artifacts of both, sleep and wake EEG, were identified semi-automatically. Epochs were excluded whenever power in the beta (20–40 Hz) and delta (0.8–4.6 Hz) band exceeded a threshold based on a moving average determined over twenty 30-s epochs [27]. A detailed analysis of the sleep spindle peak height and frequency was performed. Individual peaks in the spindle frequency range (10 to 15 Hz) were determined visually and spindle peak height was calculated as height of the individual peak minus background activity [27], [28]. Further analysis of the wake alpha peak frequency was performed in a similar manner as the spindle peak analysis.

### Statistical analysis

#### Effect of altitude and acetazolamide treatment

**Figure 2:**
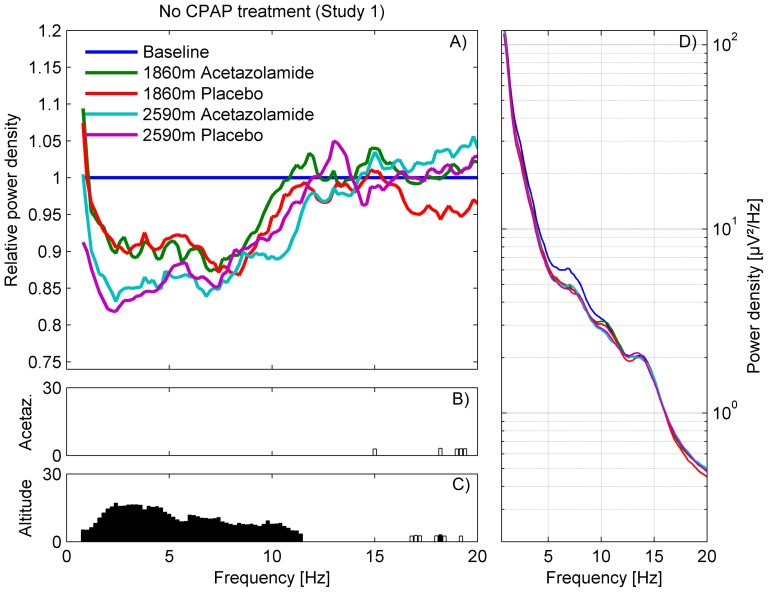
Non-REM sleep EEG power density spectra of study 1 (no CPAP treatment [16]): Moderate altitude compared to baseline. A) Average spectra (n = 39) at moderate altitude (1860 m and 2590 m) of the acetazolamide (250 mg in the evening) and placebo condition are plotted relative to baseline sleep (490 m; line at 1). Frequency resolution: 0.2 Hz. B and C) F-values of the frequency bins with significant effect of factor *Treatment* and *Altitude* of mixed model ANOVA with factors *Altitude, Treatment* and *Order of treatment*. Bins, which showed a trend, are indicated with white bars. D) Average absolute non-REM sleep EEG power density spectra of the 5 nights.

**Figure 3:**
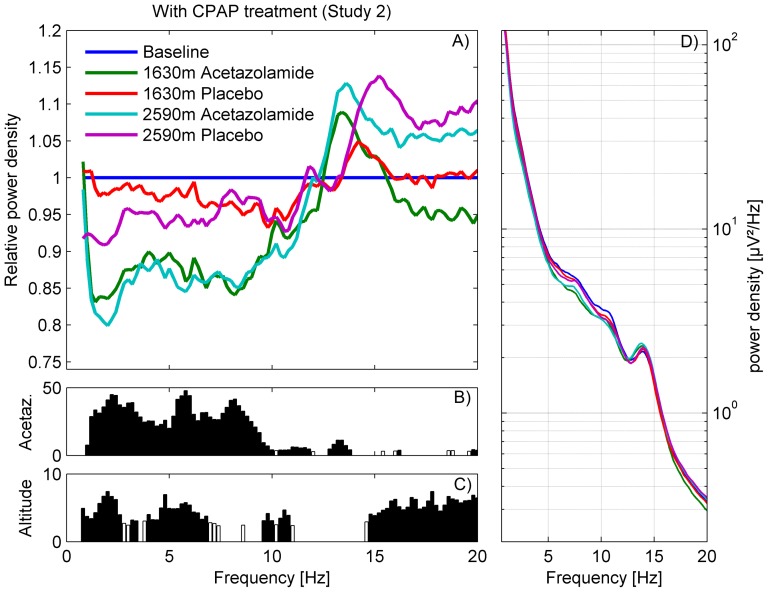
Non-REM sleep EEG power density spectra of study 2 (with CPAP treatment [3]): Moderate altitude compared to baseline. A) Average spectra (n = 41) at moderate altitude (1630 m and 2590 m) of the acetazolamide (500 mg in the evening) and placebo condition are plotted relative to baseline sleep (490 m; line at 1). Frequency resolution: 0.2 Hz. B and C) F-values of the frequency bins with significant effect of factor *Treatment* and *Altitude* of mixed model ANOVA with factors *Altitude, Treatment* and *Order of treatment*. Bins, which showed a trend, are indicated with white bars. D) Absolute non-REM sleep EEG power density spectra of the 5 nights.

Sleep and respiratory variables for study 1 and 2 were summarized as medians and interquartile ranges (Table S1 and S2 in File S1). Comparisons between measurements at altitude and baseline and the effect of acetazolamide compared to placebo were examined by Wilcoxon signed rank tests. To investigate the impact of altitude and treatment (placebo vs. acetazolamide) on the non-REM sleep EEG spectra, a linear mixed model ANOVA with factors *Altitude* [490 m, 1860 m resp. 1630 m and 2590 m], *Treatment* [acetazolamide or placebo], *Order of Treatment* [acetazolamide-placebo, placebo-acetazolamide] and their interaction was performed per frequency bin (Figure 2 and 3).

#### Effect of treatment with acetazolamide compared to placebo

**Figure 4:**
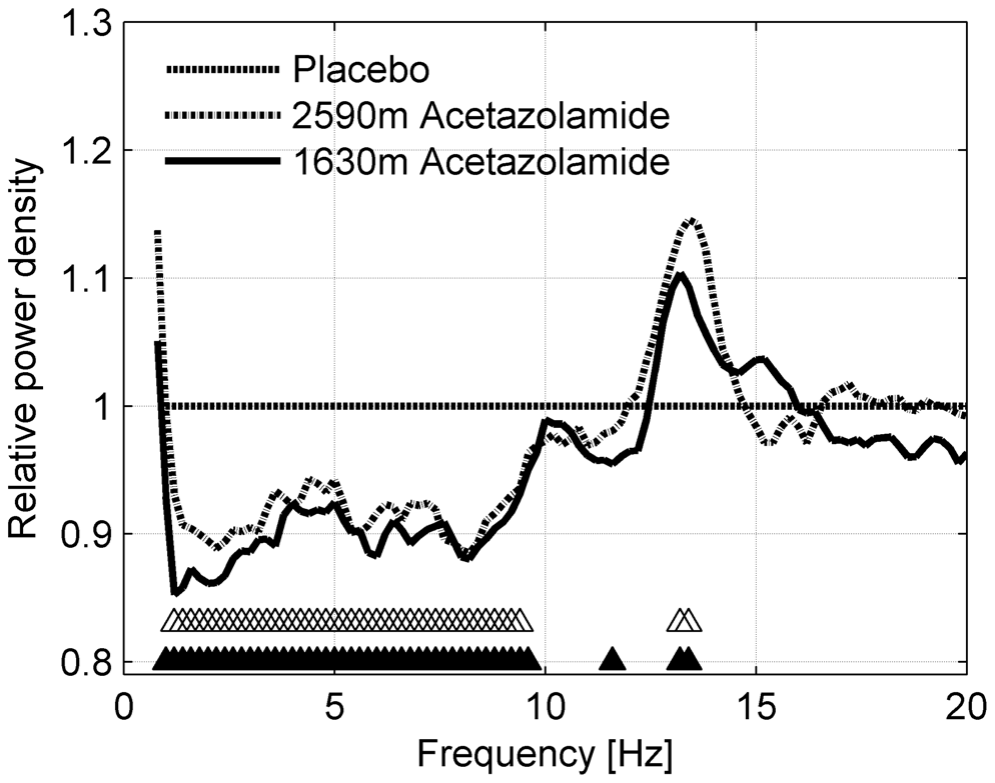
Effect of acetazolamide on non-REM sleep EEG spectra compared to placebo at 1630 m and 2590 m (Study 2). Non-REM sleep EEG power density spectra of the acetazolamide conditions (500 mg in the evening) at moderate altitude (1630 m and 2590 m) are plotted relative to the placebo conditions at the corresponding altitude (line at 1). Significant differences (p<0.05, paired t-test) between acetazolamide and placebo are indicated by “▵” for 2590 m and “▴” for 1630 m (n = 39). Frequency resolution: 0.2 Hz.

Since the mixed model ANOVA with factors *Altitude* and *Treatment* revealed an effect of treatment in study 2 (Figure 3), we further investigated the impact of acetazolamide on the non-REM sleep EEG in this study. Relative spectra of acetazolamide compared to placebo at both altitudes (acetazolamide/placebo at 1630 m and acetazolamide/placebo at 2590 m) are shown in Figure 4. Differences between conditions were investigated by bin-wise paired t-tests. In addition, visually detected spindle peak characteristics (frequency and height) were compared between acetazolamide and placebo conditions by paired t-tests.

**Figure 5:**
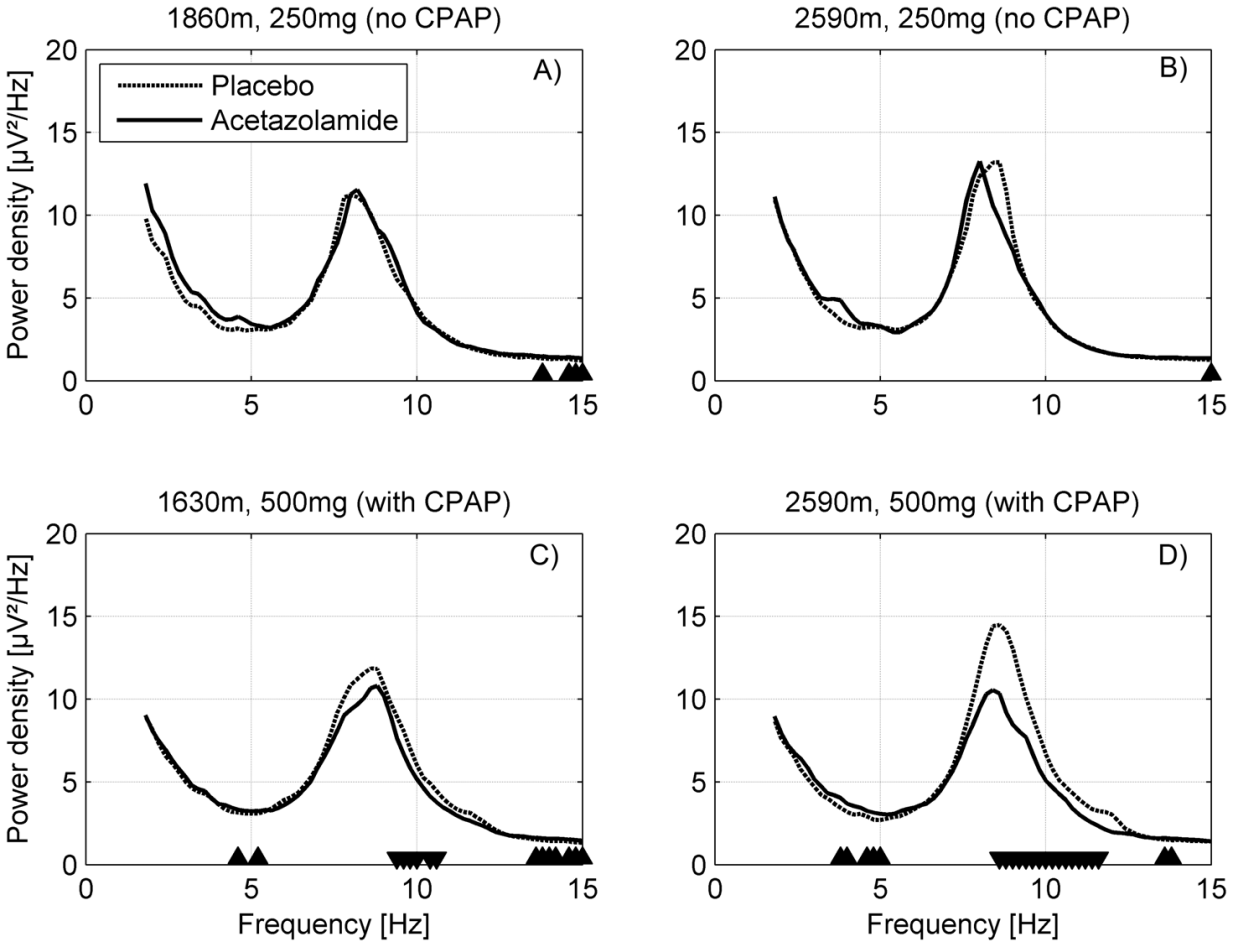
Effect of acetazolamide on wake EEG spectra during the sleep episode. In study 1 (**A and B**; ‘no CPAP treatment’; n = 30) 250 mg acetazolamide were administered in the evening and in study 2 (**C and D**; ‘with CPAP treatment’; n = 33) 500 mg. Acetazolamide was compared to placebo at both altitudes. “▴” Increase in spectral power acetazolamide compared to placebo. “▾” Decrease in spectral power acetazolamide compared to placebo (p<0.05 paired t-test).

Similar to the sleep EEG analysis, wake EEG spectra of acetazolamide and placebo conditions at both altitudes were compared by bin-wise paired t-tests and wake alpha peak frequencies of study 1 and 2 were compared by paired t-tests at both altitudes (Figure 5).

As single frequency bins may reach significance by chance but would not be clustered in a band, we considered it relevant for our interpretation only if ≥5 consecutive frequency bins (a range of 1.0 Hz) showed a significant change.

#### Effect of short-term CPAP discontinuation

**Figure 6:**
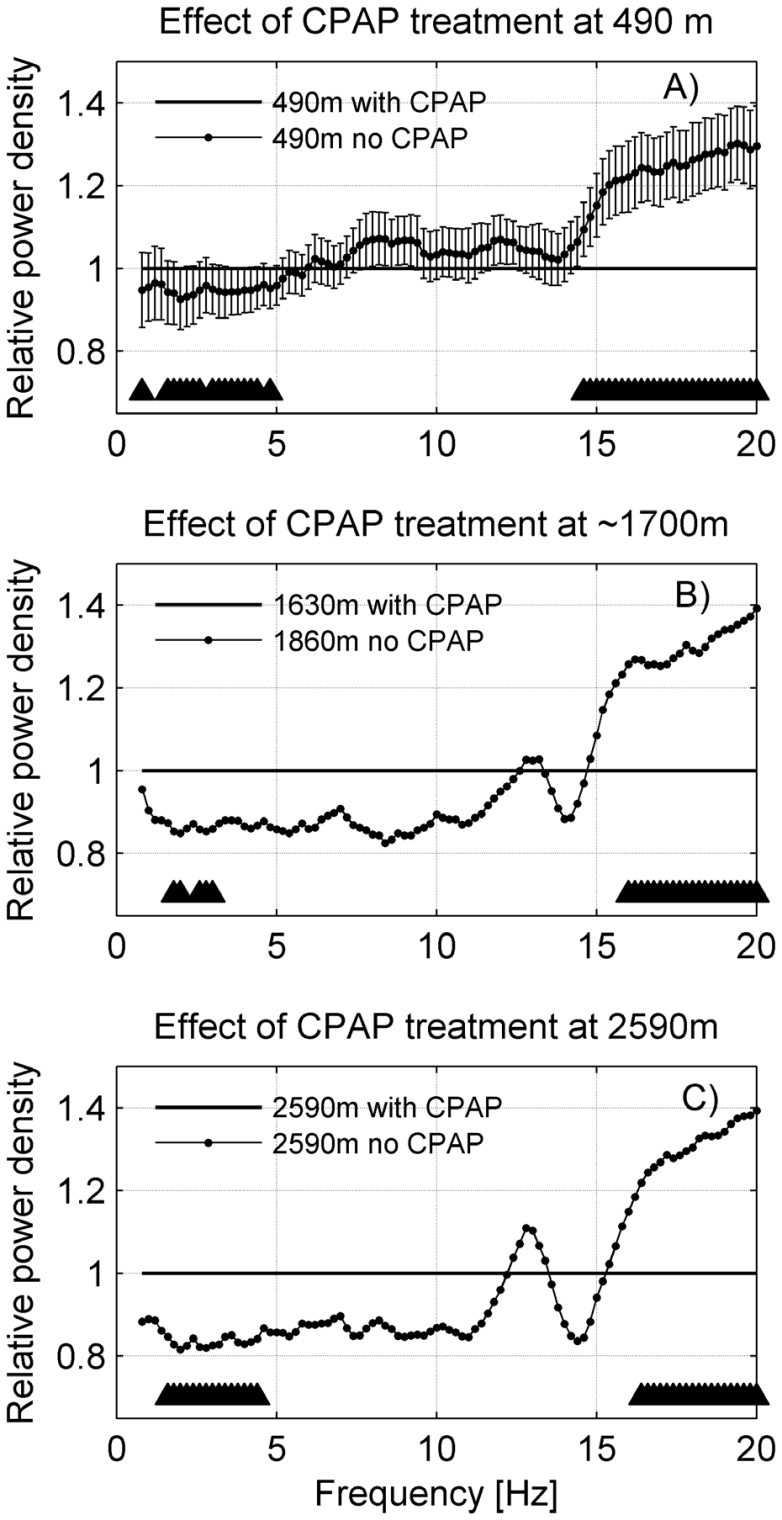
Impact of CPAP treatment on non-REM sleep EEG spectra at 490 m and at moderate altitudes. **A**) Data of study 1. Non-REM sleep EEG power density spectra of 41 OSAS patients sleeping at 490 m ‘with CPAP’ (line at 1; AHI = 6.3 [1/h]) compared to sleeping during interrupted CPAP treatment (‘no CPAP’; AHI = 58.3 [1/h]). “▴” p<0.05 paired t-test comparing CPAP to ‘no CPAP’. **B**) Non-REM sleep EEG power density spectra of OSAS patients during ‘no CPAP’ (study 1, 1860 m placebo condition; n = 39) compared to ‘with CPAP’ (line at 1; study 2, 1630 m placebo condition; n = 41) and **C**) non-REM sleep EEG power density spectra of OSAS patients during ‘no CPAP’ (study 1, 2590 m placebo condition) compared to ‘with CPAP’ (line at 1; study 2, 2590 m placebo condition). “▴” p<0.05 unpaired t-test ‘with CPAP’ compared to ‘no CPAP’.

We investigated the impact of short term CPAP discontinuation on the non-REM sleep EEG at baseline and both moderate altitudes (Figure 6). The effect of CPAP discontinuation was examined A) at 490 m by a within subject comparison (bin-wise paired t-tests) of the two recordings at 490 m performed in study 2; by a between subject comparison (bin-wise unpaired t-test): B) CPAP treatment (placebo condition at 1630 m, study 2) vs. no CPAP treatment (placebo condition at 1860 m, study 1) and C) CPAP treatment (placebo condition at 2590 m, study 2) vs. no CPAP treatment (placebo condition at 2590 m, study 1). Again, only if ≥5 consecutive frequency bins showed a significant change were they considered relevant.

## Results

### Effect of acetazolamide on non-REM sleep and respiratory variables

Similar to the previous analysis of the full dataset [3], [22], acetazolamide reduced central apneas at altitude and increased sleep quality, oxygen saturation (SpO_2_) and carbon dioxide (CO_2_; Table S1 and Table S2 in File S1).

### Effect of 250 mg acetazolamide on non-REM sleep EEG spectra (Study 1, n = 39)

An evening dose of 250 mg acetazolamide did not affect the non-REM sleep EEG spectra. On the other hand, sleep EEG power density in the lower frequency range (0.8–11.4 Hz; Figure 2) was reduced in an altitude-dependent manner (mixed model ANOVA with factors *Altitude*, *Treatment* and *Order of Treatment*). No interactions between *Altitude* and *Treatment* were observed.

### Effect of 500 mg acetazolamide on non-REM sleep EEG spectra (Study 2, n = 41)

Spectral analysis of the non-REM sleep EEG revealed an altitude- and treatment-dependent reduction of power density in the lower frequency range (0.8–11.6 Hz; Figure 3; mixed model ANOVA with factors *Altitude*, *Treatment* and *Order of Treatment*). Acetazolamide and altitude both contributed to the reduction in the lower frequency range (*Treatment*: 1–11.8 Hz, *Altitude*: 0.8–6.8 Hz and 9.6–10.8 Hz). In addition, *Altitude* had an effect on the higher frequency range (14.8–20 Hz), while *Treatment* revealed differences at 12.8–13.8 Hz, indicating changes in the spindle frequency range (11–16 Hz; sigma activity). No interactions between *Altitude* and *Treatment* were present.

### Effects of acetazolamide compared to placebo

#### Non-REM sleep EEG spectra

Since the ANOVA revealed an effect of treatment (evening dose of 500 mg) in study 2, we further investigated the effect of acetazolamide by comparing EEG spectra of acetazolamide to placebo at each altitude (e.g. acetazolamide/placebo at 1630 m). In this way, effects that are due to altitude are present in both, placebo and acetazolamide condition. A direct comparison of the two should therefore reveal the impact of acetazolamide on the EEG spectrum independent of altitude. At both altitudes acetazolamide reduced power density in the lower frequency range from 1 to 9.6 Hz by approximately 10% compared to placebo at the corresponding altitude (Figure 4). Furthermore, power density at 13.2–13.4 Hz was increased by 10–15%. This change in the spindle frequency range (sigma activity) suggests that spindle peak characteristics may have been affected (Figure 3D, absolute spectra). Thus, we further investigated the effect of acetazolamide on the spindle peak frequency and height in a subset of 34 subjects (those that showed a spindle peak in the non-REM sleep EEG spectra; see methods). Acetazolamide slightly reduced the frequency of the spindle peak (1630 m: 0.1 Hz, p<0.05; 2590 m: 0.2 Hz, p<0.001) and increased peak height (1630 m: 10%, p<0.01; 2590 m: 9%, p<0.1).

#### Wake EEG spectra

Similar to the non-REM sleep EEG, the wake EEG spectra (after lights off) were only affected after administration of an evening dose of 500 mg acetazolamide (study 2; Figure 5). Irrespective of altitude power in the 3.8–5.2 Hz and 13.6–15 Hz range was increased by acetazolamide (Figure 5C and D) whereas alpha power (8.6–11.6 Hz) was reduced compared to placebo. Since the change in the alpha range was not centered on the alpha peak, we suspected that administration of acetazolamide led to a shift in the alpha peak frequency. Additional analysis of the alpha peak frequency confirmed that similar to the spindle peak, the location of the peak was slightly shifted to lower frequencies by administration of acetazolamide (2650 m: 0.3 Hz, p<0.05; 1630 m: 0.2 Hz, p<0.1).

### Effects short-term discontinuation of CPAP treatment

The effect of short-term CPAP discontinuation on the non-REM sleep EEG was investigated at 490 m and both moderate altitudes (Figure 6). Within subject comparison were only possible at 490 m where the subjects of study 2 were recorded with and without CPAP treatment (see methods). Abstaining from CPAP during one night resulted in reduced power in the delta range (0.8–4.8 Hz; approximately 5%) and increased power in the beta range (14.6–20 Hz; approximately 25%; Figure 6).

## Discussion

This is the first study examining the effect of two doses of acetazolamide on cortical activity in OSAS patients with and without CPAP treatment. Compared to placebo, acetazolamide reduced non-REM sleep EEG spectral power in the lower frequency range (1 to 9.6 Hz) and increased sleep spindle (sigma) activity independent of altitude. In addition, acetazolamide slightly slowed the frequency of the spindle peak in non-REM sleep EEG spectra and the alpha peak in waking EEG spectra. Marked changes in brain activity were also observed at baseline and both moderate altitudes as a result of discontinued CPAP treatment. Short-term discontinuation of CPAP treatment for a few nights reduced delta power (0.8–4.8 Hz) and increased beta power (14.6–20 Hz) most likely due to the occurrence of obstructive apneas.

### Acetazolamide compared to placebo

Acetazolamide is a well-established and frequently used drug to treat acute mountain sickness (AMS) [19] and associated high-altitude periodic breathing [24]. In addition, acetazolamide is used to treat patients suffering from central sleep apnea due to heart failure [29]. The recommended dosage of acetazolamide is 250–750 mg per day [30]. In the present two studies subjects received 500 mg in study 1 (250 mg in the morning and evening [22]) and 750 mg in study 2 (250 mg in the morning, 500 mg in the evening [3]). While the dose administered in the morning was 250 mg in both studies, the evening-dose was twice as high in study 2 compared to study 1 (500 mg vs. 250 mg). Since the half-life time of acetazolamide is 6–9 hours [31], one can assume that the morning-dose had minimal influence at night and the main effect on sleep and breathing variables was due to evening doses. Independent of the dose, acetazolamide reduced the total AHI, by abolishing central apneas, increased SpO_2_ and reduced PCO_2_ in both studies [3], [22]. In terms of sleep architecture, waking was reduced and percent of non-REM sleep and sleep efficiency were increased with acetazolamide. Changes in cortical activity as investigated by spectral analysis of the sleep and waking EEG were, however, only observed with the evening-dose of 500 mg in study 2.

The reduction of EEG power in the lower frequency range (1 to 9.6 Hz) and increase in spindle power after treatment with acetazolamide is very similar to the sleep EEG changes observed after administration of Benzodiazepines or analogs (Z-drugs) [32]–[35]. Binding of Benzodiazepines to brain GABA_A_ receptors promotes binding of GABA and leads to hyperpolarization of the cells. Benzodiazepines thereby reduce the excitability of neurons [36]. The GABA_A_ ionophore is selectively permeable to chloride (Cl^-^) and bicarbonate (HCO_3_^-^) [37]. Opening of the membrane channel by GABA leads to a slight efflux of HCO_3_^-^ and influx of Cl^-^, resulting in a more negative membrane potential [25]. Similar to Benzodiazepines, acetazolamide might lead to an increased efficacy of GABA-mediated inhibition. As a carbonic anhydrase inhibitor, acetazolamide leads to renal bicarbonate excretion and limits the HCO_3_^-^ efflux from the GABA channel [25]. We therefore attribute the benzodiazepine-like effect (spectral “fingerprint”) of acetazolamide on the sleep EEG to reduced excitability of neurons due to GABAergic hyperpolarization induced by a reduction in bicarbonate concentration. Due to their inhibiting effect on neuronal excitability both, acetazolamide and Benzodiazepines, are also effective as anticonvulsants in epileptic patients [25], [36]. The mechanisms explaining how increased GABAergic inhibition leads to the spectral “fingerprint” of Benzodiazepines, however remain unknown.

Both doses of acetazolamide were, however, sufficient to reduce the central AHI and stabilize sleep, but only the higher evening-dose (500 mg) revealed changes in brain oscillations observed with spectral analysis. Whether the changes in brain oscillations observed at the higher dose are beneficial or detrimental is unclear. On the one hand, the absence of changes to cortical oscillations of the lower dose may be advantageous as cortical oscillations remain unchanged. On the other hand, benzodiazepines and analogs are used to improve sleep quality and the benzodiazepine-like effect of the higher dose on cortical oscillations may positively impact sleep. Future studies should investigate the dose-dependent effect of acetazolamide on not only cortical oscillations but also other outcomes measures, such as behavioral testing (e.g. daytime alertness), to reach a final conclusion about the best dosage.

### Short-term discontinuation of CPAP therapy

Treatment with CPAP improves sleep quality [3], [4], [38] and reduces daytime symptoms [5], [6], [39]. For example slow-wave and REM sleep duration as well as sleep efficiency are increased with CPAP treatment and in turn the duration of stage 1 sleep as well as the number of arousals is reduced [3], [4], [6], [38]. In addition, daytime sleepiness (as measured by MSLT), cognitive performance and mood were improved after several weeks of CPAP treatment [5], [6], [39]. In contrast to the aforementioned studies which examined previously untreated patients, we measured the impact of short-term discontinuation of CPAP treatment. We observed that independent of altitude (490 m, 1630/1860 m and 2590 m) non-REM sleep slow-wave activity (SWA; 0.8–4.6 Hz) was reduced and beta activity increased, if OSAS patients discontinued their CPAP treatment for a few days. This observation is in agreement with the reduction in SWA and increase in beta activity observed during epochs of stage 2 sleep containing a respiratory disturbance in healthy subjects sleeping at moderate altitude [40]. A reduction in SWA and increase in beta activity was also observed in untreated OSAS patients compared to healthy controls [9]. We therefore attribute the alterations in brain oscillations observed during a night where patients discontinued CPAP treatment mainly to be due to respiratory disturbances and subsequent micro arousals or EEG activation, while other factors such as fluctuations in oxygen saturation may have only minor effects on EEG activity.

SWA is considered a reliable indicator of sleep depth or sleep intensity and is used to measure sleep homeostasis [41]. Suppression of SWA due to sleep disturbances, sleep restriction or sleep deprivation leads to increased daytime sleepiness and impaired performance [42], [43]. The impact of CPAP treatment on SWA in sleep apnea patients was investigated by Heinzer et al. [12]. They observed an increase in mean SWA after 9 months of CPAP treatment concurrent with a restoration of the physiologic decay of SWA across the night. In the same study, SWA in untreated patients was positively correlated with daytime sleepiness as assessed by MSLT. Even short-term discontinuation from CPAP treatment may therefore have adverse effects on daytime functioning of patients suffering from OSAS.

### The effect of altitude on the sleep EEG spectra of OSAS patients

A consistent altitude-effect on the non-REM sleep EEG spectra is in agreement with previous findings in healthy subjects [27]. Power in the lower frequency range (0.8–11.6 Hz) was reduced in an altitude-dependent manner, independent of CPAP treatment. These changes are further discussed in Discussion S1 in the File S1.

## Conclusions

In summary, using quantitative analysis of the EEG as a measure of cortical oscillations, we show that acetazolamide affects cortical oscillations during sleep and wakefulness in a dose-dependent manner. We demonstrated that discontinuation of CPAP treatment has immediate adverse effects on SWA, which is known to reflect the restorative functions of sleep. Last, we confirmed a reduction in SWA with increasing altitude in patients suffering from OSAS as previously observed in healthy subjects.

## Supporting Information

File S1 Table S1. Sleep and respiratory variables in study 1 [1]. OSAS patients withdrawn from CPAP treatment. *p<0.05, compared to 490 m (Wilcoxon signed ranks test). ‡p<0.05, compared to corresponding placebo condition at the same altitude (Wilcoxon signed ranks test). Data are shown as medians and interquartile ranges. AHI: Apnea/Hypopnea Index; SpO_2_: Oxygen saturation; etCO_2_: End tidal carbon dioxide pressure; REMS: Rapid-eye movement sleep; SWS: Slow-wave sleep; TST: Total sleep time; TIB: Time in bed; Sleep efficiency. TST as percentage of TIB. N = 39. Table S2. Sleep and respiratory variables in study 2 [2]. OSAS patients treated with CPAP. *p<0.05, compared to 490 m with CPAP (Wilcoxon signed ranks). ‡p<0.05, compared to corresponding placebo condition at the same altitude (Wilcoxon signed ranks). Data are shown as medians and interquartile ranges. AHI: Apnea/Hypopnea Index; SpO_2_: Oxygen saturation; PtcCO_2_: Transcutaneous carbon dioxide pressure; REMS: Rapid-eye movement sleep; SWS: Slow-wave sleep; TST: Total sleep time; TIB: Time in bed; Sleep efficiency. TST as percentage of TIB. N = 41. Discussion S1. Supporting discussion. References S1. Supporting references.
(DOCX)
