# Supplementary material for: Impact of Acetazolamide and CPAP on Cortical Activity in Obstructive Sleep Apnea Patients
Source: PLoS One. 2014 Apr 7;9(4):e93931. doi: 10.1371/journal.pone.0093931 (PMC3977962; doi:10.1371/journal.pone.0093931)
Supplement: File S1 — Table S1. Sleep and respiratory variables in study 1 [1]. OSAS patients withdrawn from CPAP treatment. *p<0.05, compared to 490 m (Wilcoxon signed ranks test). ‡p<0.05, compared to corresponding placebo condition at the same altitude (Wilcoxon signed ranks test). Data are shown as medians and interquartile ranges. AHI: Apnea/Hypopnea Index; SpO2: Oxygen saturation; etCO2: End tidal carbon dioxide pressure; REMS: Rapid-eye movement sleep; SWS: Slow-wave sleep; TST: Total sleep time; TIB: Time in bed; Sleep efficiency. TST as percentage of TIB. N = 39. Table S2. Sleep and respiratory variables in study 2 [2]. OSAS patients treated with CPAP. *p<0.05, compared to 490 m with CPAP (Wilcoxon signed ranks). ‡p<0.05, compared to corresponding placebo condition at the same altitude (Wilcoxon signed ranks). Data are shown as medians and interquartile ranges. AHI: Apnea/Hypopnea Index; SpO2: Oxygen saturation; PtcCO2: Transcutaneous carbon dioxide pressure; REMS: Rapid-eye movement sleep; SWS: Slow-wave sleep; TST: Total sleep time; TIB: Time in bed; Sleep efficiency. TST as percentage of TIB. N = 41. Discussion S1. Supporting discussion. References S1. Supporting references. (DOCX) [file pone.0093931.s001.docx]

**SUPPORTING INFORMATION**

**Table S1.** Sleep and respiratory variables in study 1 [1]. OSAS patients withdrawn from CPAP treatment.

|  | 490 m | 1860m  Placebo | 1860m Acetazolamide | 2590m  Placebo | 2590m Acetazolamide |
| --- | --- | --- | --- | --- | --- |
| **Total AHI**  **(1/h)** | 53.9  [42.9; 72.1] | 65.0  [50; 65] | 48.0‡  [26.2; 64.9] | 87.3*  [67.9; 105.1] | 62.2‡  [51.9; 84.1] |
| **Central**  **AHI (1/h)** | 1.5  [0.4; 2.3] | 16.3*  [8.2; 24.5] | 8.3*‡  [4.2; 14.2] | 23.3*  [14.0; 43.1] | 4.9*‡  [1.1; 13.8] |
| **Obstructive AHI (1/h)** | 50.9  [42.3; 68.5] | 42.5  [26.2; 72.1] | 39.0*  [13.4; 54.2] | 56.2  [38.9;75.0] | 54.0  [39.4; 76.3] |
| **SpO_2_**  **(%)** | 93  [92; 94] | 89  [88; 90] | 91*‡  [89; 93] | 85  [83; 88]* | 88*‡  [85; 89] |
| **etCO_2_**  **(mmHg)** | 50  [49; 50] | 44*  [42; 46] | 39*‡  [36.3; 43] | 38*  [34.3; 39] | 36*‡  [33; 39] |
| **Sleep efficiency (%)** | 78.4  [61.7; 85.5] | 75.8  [66; 81.7] | 81.8*‡  [71.2; 86.5] | 70.9  [52.9; 82.9] | 76.9‡  [63.2; 85.3] |
| **non-REMS**  **(% TST)** | 92  [87.4; 97] | 89.4*  [83.4; 93.3] | 93.1‡  [86.2; 97.5] | 90.8  [83.8; 99.2] | 90.7  [87.4; 97.7] |
| **REMS**  **(% TST)** | 8  [3; 12.6] | 10.6*  [6.7; 16.6] | 6.9  [2.7; 13.8] | 7.9  [1.5; 16.5] | 7.5  [2.6; 11.8] |
| **S1+S2**  **(% TST)** | 86.4  [74.2; 92.2] | 79.8*  [69.5; 87.7] | 85.9  [71.2; 92.6] | 84.9  [76.8; 93.1] | 87.4  [79.8; 92.9] |
| **SWS**  **(% TST)** | 6.6  [1.9; 14.9] | 9.5  [2.6; 15.9] | 8.3  [1.3; 13.7] | 3.5*  [0; 8.7] | 3.4*  [0; 10.4] |
| **Wake**  **(% TIB)** | 21.6  [14.5; 38.3] | 24.2  [18.3; 35.6] | 17.9*‡  [13.5; 27.4] | 34  [17.7; 47.3] | 22.9‡  [14.7; 32.3] |
| **Arousals**  **(1/h)** | 47.2  [39.2; 67.8] | 61.6  [42.3; 76.8] | 48.7‡  [28.7; 64.5] | 73.3*  [56.5; 85] | 55.1‡  [43.0; 82.7] |

**Table S2.** Sleep and respiratory variables in study 2 [2]. OSAS patients treated with CPAP.

|  | **490 m**  **without CPAP** | **490 m with CPAP** | **1630 m Placebo** | **1630 m Acetazolamide** | **2590 m Placebo** | **2590 m Acetazolamide** |
| --- | --- | --- | --- | --- | --- | --- |
| **Total AHI (1/h)** | 58.3*  [38.4; 74.7] | 6.3  [4.6; 11] | 11.1*  [5.3; 20.1] | 5.8‡  [2.9; 10.1] | 19.3*  [9.2; 26.6] | 6.9‡  [3.8; 10.1] |
| **Central AHI**  **(1/h)** | 0.9  [0.4; 2.9] | 1.6  [0.5; 3.8] | 4.3*  [1.2; 14.9] | 1.8‡  [0.7; 5.2] | 10.8*  [5.3; 21.3] | 4‡*  [1.2; 7.3] |
| **Obstructive AHI (1/h)** | 56.1*  [36.3; 73.8] | 4.2  [2.3; 7.2] | 4  [1.6; 7.4] | 2.2‡*  [0.8; 3.9] | 3.1  [1.6;8] | 2.3*  [1; 5.8] |
| **SpO_2_**  **(%)** | 93*  [92; 94] | 95  [94; 96] | 93*  [92; 94] | 94‡*  [93; 95] | 89*  [87.8; 91] | 91‡*  [90; 92] |
| **PtcCO_2_ (mmHg)** | 47  [43.9; 50] | 47  [42.5; 51] | 44  [41.5; 48] | 42.5*‡  [38; 45.6] | 43.5*  [40; 46.3] | 39.5‡*  [37; 42.6] |
| **Sleep efficiency (%)** | 81  [74; 90] | 82.4  [75; 90] | 83  [78; 90] | 88.2*‡  [80; 92] | 79  [69; 88] | 86.8‡*  [83; 92] |
| **non-REMS**  **(% TST)** | 85.2*  [80.8; 91] | 81.3  [76.1; 85.7] | 75.6*  [72.4; 81.5] | 80.6‡  [77.6; 84.3] | 82.6  [77.4; 86] | 83.2‡  [79.2; 87.3] |
| **REMS**  **(% TST)** | 14.8*  [9; 19.2] | 18.7  [14.3; 23.9] | 24.4*  [18.5; 27.6] | 19.4‡  [15.7; 22.4] | 17.4  [14; 22.6] | 16.8‡  [12.7; 20.8] |
| **S1+S2**  **(% TST)** | 72.1*  [64.8; 84.2] | 65.3  [59.2; 73.7] | 63.9  [57.8; 70.1] | 66.4  [60.7; 74.3] | 71.6*  [64.5; 75.7] | 70.6*  [64.9; 76.9] |
| **SWS**  **(% TST)** | 11.2*  [4.6; 16.9] | 15.1  [10; 21] | 12.5*  [8.6; 17.1] | 15  [9.3; 17.4] | 10.4*  [7.5; 14.1] | 12.5‡*  [8.5; 17.3] |
| **Wake**  **(% TIB)** | 16.5  [9.1; 26.2] | 17.1  [9.7; 25.1] | 16.5  [9.9; 21.8] | 11.1*‡  [7.3; 18.6] | 20.8  [10.9; 30.5] | 12.9*‡  [7.8; 16.9] |
| **Arousals**  **(1/h)** | 37.9*  [26.3; 49.8] | 15.4  [11.1; 28.7] | 17.5  [12; 29.3] | 18  [13.2; 25.3] | 15.2  [11.1; 25.6] | 14.2  [11; 24.5] |

**Discussion S1**

*Moderate altitude compared to baseline*

Both studies (study 1 without and study 2 with CPAP treatment) revealed an altitude effect on the lower frequency range of the non-REM sleep EEG. The reduction in spectral power in the range of 1-10 Hz was more pronounced in patients, which were not treated with CPAP. An explanation might be that alterations in brain activity during discontinued CPAP treatment as described above, lead to a brain state, which is more sensitive to altitude. However, the impact of pressure applied during CPAP treatment (5-15 cm H_2_O[2], equivalent to 4-11 mm Hg) might minimally reduce the effect of altitude (718 mm Hg at 490 m; 562 mm Hg at 2590 m), but most likely not enough to be relevant in the present context.

Similar changes in the non-REM sleep EEG have been observed in a previous study on 44 healthy subjects sleeping at 1630 m and 2590 m [3,4]. The reduction in power in the lower frequency range of the non-REM sleep EEG therefore seems to be a general effect during sleep at altitude. Moreover, in both, healthy subjects and OSAS patients the effect was altitude-dependent and increased with increasing altitude.

**References S1**

1. Nussbaumer-Ochsner Y, Ursprung J, Siebenmann C, Maggiorini M, Bloch KE (2012) Effect of short-term acclimatization to high altitude on sleep and nocturnal breathing. Sleep 35: 419-423.

2. Latshang TD, Nussbaumer-Ochsner Y, Henn RM, Ulrich S, Lo Cascio CM, et al. (2012) Effect of acetazolamide and autoCPAP therapy on breathing disturbances among patients with obstructive sleep apnea syndrome who travel to altitude: a randomized controlled trial. Jama 308: 2390-2398.

3. Stadelmann K, Latshang TD, Lo Cascio CM, Tesler N, Stoewhas AC, et al. (2013) Quantitative changes in the sleep EEG at moderate altitude (1630 m and 2590 m). PLoS One 8: e76945.

4. Stadelmann K, Latshang TD, Tarokh L, Lo Cascio CM, Tesler N, et al. (2014) Sleep respiratory disturbances and arousals at moderate altitude have overlapping EEG spectral signatures. J Sleep Res (in press).
